# Early Changes in Renal Function as Predictors of In-Hospital Mortality in COVID-19 Patients

**DOI:** 10.3390/life16020331

**Published:** 2026-02-14

**Authors:** Nicu Olariu, Nilima Rajpal Kundnani, Simona Ruxanda Dragan, Luciana-Elena Marc, Victor Buciu, Delia Mira Berceanu Vaduva, Andreas Valcovici, Ioana Adela Ratiu, Petru Bucuras, Adelina Mihaescu

**Affiliations:** 1 Department of Internal Medicine II, Division of Nephrology, “Victor Babeș” University of Medicine and Pharmacy, 300041 Timisoara, Romania; nicu.olariu@umft.ro (N.O.); mihaescu.adelina@umft.ro (A.M.); 2 Center for Molecular Research in Nephrology and Vascular Disease, Faculty of Medicine, “Victor Babeș” University of Medicine and Pharmacy, 300041 Timisoara, Romania; 3 Doctoral School, “Victor Babeș” University of Medicine and Pharmacy, 300041 Timisoara, Romania; 4 University Clinic of Internal Medicine and Ambulatory Care, Prevention and Cardiovascular Recovery, Department VI—Cardiology, “Victor Babeș” University of Medicine and Pharmacy, 300041 Timisoara, Romania; simona.dragan@umft.ro; 5 Research Centre of Timisoara, Institute of Cardiovascular Diseases, “Victor Babeș” University of Medicine and Pharmacy, 300041 Timisoara, Romania; 6 Department XIV—Microbiology, “Victor Babeș” University of Medicine and Pharmacy, 300041 Timisoara, Romania; 7 Faculty of Medicine and Pharmacy, University of Oradea, 1st December Square 10, 410073 Oradea, Romania; 8 Nephrology Department, Emergency Clinical Hospital Bihor County, 12 Corneliu Coposu Street, 410073 Oradea, Romania; 9 Nephrology Clinic, “Pius Brinzeu” Emergency County Hospital, 300723 Timisoara, Romania

**Keywords:** acute kidney injury, COVID-19, hemodialysis, inflammation, eGFR

## Abstract

Background: Acute kidney injury (AKI) is a frequent and prognostically relevant complication of COVID-19. However, reliance on static creatinine values or binary AKI definitions may overlook clinically meaningful early renal dynamics. We evaluated whether early renal function trajectories within the first 24–48 h of hospitalization provide incremental prognostic information. Methods: We conducted a retrospective, single-center cohort study of adults hospitalized with laboratory-confirmed COVID-19 between December 2020 and December 2021. Early renal function patterns were defined using KDIGO-based changes in serum creatinine between admission and 24–48 h, classifying patients as stable, early improvement, or early deterioration. The primary outcome was in-hospital mortality. Multivariable logistic regression adjusted for age, sex, chronic kidney disease, comorbidities, inflammatory burden (C-reactive protein), nutritional status (albumin), pulmonary involvement, and treatment variables. Results: Among 721 patients, 65.2% had stable renal function, 22.5% had early improvement, and 12.3% had early deterioration. In-hospital mortality differed significantly across dynamic patterns (*p* = 0.007). Mortality was lowest in the stable group (35.1%) and higher in both early improvement (48.1%) and early deterioration (44.9%). After multivariable adjustment, early improvement remained independently associated with higher in-hospital mortality compared with stable renal function (adjusted OR 1.53, 95% CI 1.03–2.28), while early deterioration showed a directionally similar but non-significant association. Early improvement was also associated with higher AKI burden and increased need for acute de novo hemodialysis. Conclusions: Early renal function change patterns within the first 24–48 h of hospitalization carry prognostic value beyond static creatinine measures. Apparent early creatinine improvement may reflect recovery from prior injury or systemic instability rather than true renal recovery, identifying a subgroup at heightened risk. Classification based on early renal function assessment may enhance early risk stratification in hospitalized patients with COVID-19.

## 1. Introduction

Acute kidney injury (AKI) has been one of the most clinically impactful extra-pulmonary complications observed in hospitalized patients with COVID-19. Across early and later systematic reviews/meta-analyses, AKI occurs in a substantial proportion of hospitalized patients and is consistently associated with markedly worse short-term outcomes, including increased in-hospital mortality [1,2].

Beyond incidence, COVID-19-associated AKI has important implications for resource use and escalation of care, including the need for renal replacement therapy (RRT), which, in turn, is linked to very high mortality in the sickest cohorts [3,4,5]. Risk stratification data from meta-analyses also show that AKI in COVID-19 is not a random event.

Clinically, AKI in COVID-19 has been associated with older age, chronic kidney disease, cardiovascular disease, diabetes, and markers of systemic inflammation [6,7,8].

Mechanistically, renal injury appears to result from a combination of endothelial dysfunction, dysregulated inflammation, microvascular injury, hemodynamic instability, and venous congestion related to respiratory failure [9,10,11].

Clinically, kidney involvement in severe COVID-19 can present along a spectrum, ranging from an early loss of urinary concentrating ability to overt excretory failure with oliguria/anuria in the most critically ill patients. In real-world ward and ICU populations, this picture frequently coexists with profound metabolic and hemodynamic vulnerability, including morbid obesity, which can amplify respiratory compromise and venous congestion and thereby compound renal stress.

A limitation of much of the early clinical literature is that renal risk was frequently described using single time-point measurements (e.g., admission creatinine) or a binary AKI label, which can miss clinically meaningful dynamics that unfold shortly after presentation. However, several studies have shown that time-dependent changes in renal biomarkers provide superior prognostic information compared with single baseline measurements or binary AKI definitions [3,12].

From a practical standpoint, the first 24–48 h after admission represents an actionable window: early deterioration may reflect evolving multi-organ dysfunction, while early improvement may signal hemodynamic reversibility and therapeutic response. To operationalize this clinically relevant interval, we evaluated early renal function alteration using serum creatinine measured at admission and reassessed within the first 24–48 h. AKI was defined according to Kidney Disease: Improving Global Outcomes (KDIGO) criteria, which remain the internationally adopted reference framework for AKI definition and staging in clinical research and practice [13,14,15].

### Aim of Study

We aimed to determine whether early renal creatinine changes within the first 24–48 h of hospitalization are independently associated with in-hospital mortality in patients hospitalized with COVID-19 while secondarily exploring associations with acute de novo hemodialysis and length of stay. We hypothesized that early worsening of renal function would be associated with higher in-hospital mortality compared with stable or improving dynamic functional change, even after adjustment for demographic factors, baseline comorbidities, markers of COVID-19 severity, and inflammatory status.

## 2. Methodology of Study

### 2.1. Study Design and Population

This was a retrospective, observational, single-center cohort study conducted in a tertiary-care hospital, including consecutive adult patients hospitalized with laboratory-confirmed SARS-CoV-2 infection between December 2020 and December 2021. The study was designed and is reported in accordance with the STROBE recommendations for observational studies [16].

Eligibility for the primary analysis required the availability of serum creatinine measurements at hospital admission and within the first 24–48 h of hospitalization, allowing for classification of early renal function trajectories. The creatinine value at 24–48 h was defined as the first available serum creatinine measurement recorded within this interval. Patients with end-stage kidney disease requiring chronic dialysis prior to admission were excluded. Patients without a follow-up creatinine measurement in the 24–48 h window were not included in the early creatinine changes-based analyses but were retained for descriptive analyses to assess potential selection bias. All analyses were limited to in-hospital outcomes. Patients without repeat creatinine measurements within the first 48 h were excluded, most commonly due to early discharge, transfer, or early death.

Clinical data were collected retrospectively from electronic health records. Variables included demographic characteristics (age in years, sex), pre-existing comorbidities (chronic kidney disease, hypertension, diabetes mellitus, coronary artery disease, atrial fibrillation), and markers of COVID-19 severity (extent of pulmonary involvement on imaging).

Laboratory parameters comprised serum creatinine (mg/dL) and estimated glomerular filtration rate (eGFR, mL/min/1.73 m^2^) measured at hospital admission, at 24–48 h, and at discharge, as well as urea (mg/dL), serum sodium (mmol/L) and potassium (mmol/L), hemoglobin (g/dL), albumin (g/dL), C-reactive protein (mg/L), and procalcitonin (ng/mL). Acute kidney injury was defined according to KDIGO criteria.

Early creatinine change patterns were defined based on changes in serum creatinine between hospital admission and the 24–48 h time point, in accordance with KDIGO criteria [13]. Patients were classified into three groups: early deterioration, defined as an increase in serum creatinine of ≥0.3 mg/dL or ≥1.5 times the admission value within 48 h; stable renal function, defined as changes not meeting criteria for either deterioration or improvement; and early improvement, defined as a decrease in serum creatinine of ≥0.3 mg/dL or a reduction to ≤0.67 times the admission value within the same interval. Baseline CKD status was included as a covariate to partially account for differences in baseline renal function.

Treatment-related variables included administration of antiviral therapy (remdesivir or favipiravir), antibiotic use, and the need for acute de novo hemodialysis during hospitalization. Clinical outcomes included in-hospital mortality and length of hospital stay.

In-hospital outcomes were assessed from admission until discharge or death (i.e., in-hospital follow-up only).

Kidney-related definitions were standardized a priori. Acute kidney injury (AKI) was defined according to KDIGO criteria, which represent the internationally adopted reference framework for AKI definition and staging in both clinical research and practice [14,15]. Estimated glomerular filtration rate (eGFR) values were available in the dataset as automatically calculated laboratory outputs, consistent with routine use of the CKD-EPI creatinine equation in clinical practice. The CKD-EPI creatinine equation is widely validated and commonly implemented in hospital laboratory systems [17,18]. Urine output criteria were unavailable and therefore not included in KDIGO AKI classification, which may have led to underestimation of AKI incidence.

Patients excluded due to missing 24–48 h creatinine measurements most commonly represented individuals with very short hospital stays (early discharge, transfer, or early death) rather than systematic differences in baseline comorbidity profiles. Available admission data did not suggest major demographic or comorbidity imbalances between included and excluded patients, supporting the representativeness of the analytic cohort. Nevertheless, some degree of selection bias cannot be entirely excluded.

Data were maintained on password-protected institutional servers with access restricted to the study team. Missing data were rare (<3% for all covariates). Patients with missing creatinine levels were excluded. For other variables, complete case analysis was used, as the proportion of missingness was low and not clustered by outcome or exposure status

### 2.2. Outcomes

The primary outcome of the study was in-hospital mortality. Secondary outcomes included the initiation of acute de novo hemodialysis during hospitalization, length of hospital stay (days), and the occurrence of AKI. All outcomes were assessed exclusively during the index hospitalization.

### 2.3. Ethical Considerations

The study was conducted in accordance with the principles of the Declaration of Helsinki [19] and received approval from the local Ethics Committee (approval number 590/8 January 2026). Due to the retrospective, non-interventional nature of the study and the use of routinely collected clinical data, the requirement for individual informed consent was waived.

All data were fully anonymized prior to analysis, and data handling complied with applicable institutional policies and the General Data Protection Regulation (GDPR) [20] of the European Union. The study did not involve any intervention, modification of standard clinical care, or influence on clinical decision-making.

### 2.4. Statistical Analysis

Data entry and cleaning were performed using Microsoft Excel 2016 (Microsoft Corp., Redmond, WA, USA) [21]. Preliminary descriptive statistics and database management were carried out in SPSS Statistics version 26 (IBM Corp., Armonk, NY, USA) [22]. Advanced analyses were performed in R version 4.3.3 (R Foundation for Statistical Computing, Vienna, Austria) [23]. All statistical tests were two-sided, and a *p*-value < 0.05 was considered statistically significant.

Continuous variables were tested for normality using the Shapiro–Wilk test and are presented as mean ± standard deviation or median with interquartile range (IQR), as appropriate. Categorical variables are reported as absolute counts and percentages. Comparisons between groups defined by early renal function downfall were performed using Student’s *t*-test or one-way ANOVA for normally distributed continuous variables and the Mann–Whitney U or Kruskal–Wallis test for non-normally distributed variables. Categorical variables were compared using the chi-square test or Fisher’s exact test, as appropriate.

The association between early renal function evaluations and in-hospital mortality was evaluated using multivariable logistic regression analysis. Covariates included in the adjusted models were selected a priori based on clinical relevance and the prior literature and included age, sex, pre-existing chronic kidney disease, hypertension, diabetes mellitus, extent of pulmonary involvement, C-reactive protein at admission, serum albumin at admission, and antiviral treatment. Results are reported as odds ratios (ORs) with 95% confidence intervals (CIs).

Secondary outcomes, including acute de novo hemodialysis initiation and length of hospital stay, were analyzed using logistic regression and linear regression models, respectively, with adjustment for the same covariates when appropriate. Due to the limited number of hemodialysis events, multivariable models for this outcome were kept parsimonious. Admission creatinine was not included simultaneously with CKD status in multivariable models to avoid multicollinearity between baseline renal function indicators.

## 3. Results

### 3.1. Patient Selection

**Figure 1:**
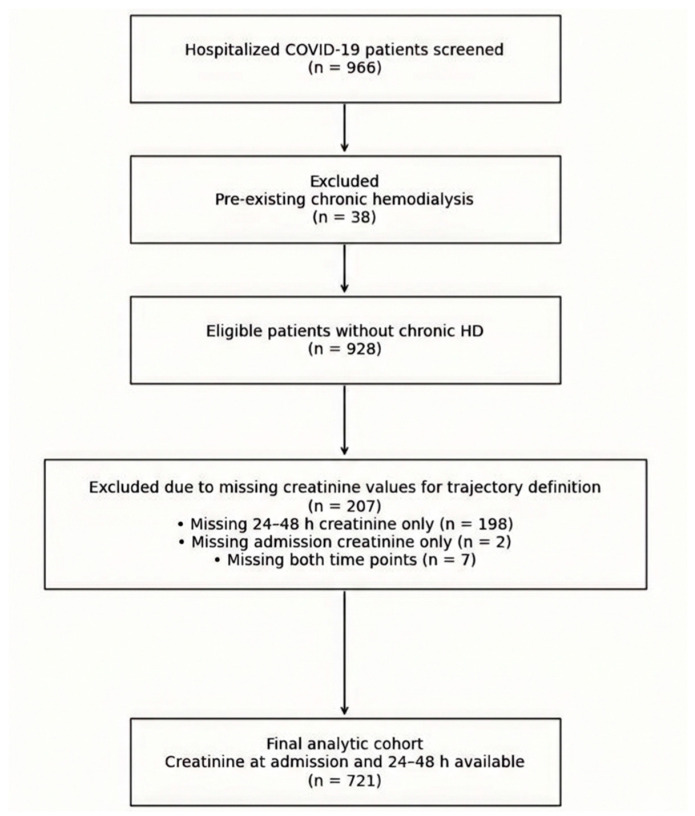
Patient selection and cohort derivation. Complete patient selection process. Data was retrospectively extracted, and patients were systematically excluded based on available information. Inclusion in the analytic cohort required complete creatinine measurements at both predefined time points used for early renal function early creatinine change assessment.

During the study period, 966 hospitalized patients with confirmed SARS-CoV-2 infection were screened. We excluded 38 patients with pre-existing chronic hemodialysis. Of the remaining 928 patients, serum creatinine at admission and within the first 24–48 h was available in 721 patients, who constituted the primary analytic cohort. Among the 207 excluded due to missing creatinine for pattern definition, 198 had admission creatinine but lacked a 24–48 h value, 2 lacked admission creatinine but had a 24–48 h value, and 7 lacked both time points. Patient selection is visualized in Figure 1.

### 3.2. Cohort Characteristics

In the primary analytic cohort (*n* = 721), the mean age was 68.0 ± 12.6 years, and 398 (55.2%) were male. Pre-existing CKD (binary variable) was present in 80 (11.1%), and KDIGO-defined AKI diagnosis (as recorded in the dataset) occurred in 176 (24.4%). The overall in-hospital mortality rate was 283 (39.3%), and 17 (2.4%) patients required acute de novo hemodialysis during hospitalization. The median length of stay was 13.0 days (IQR 7.0–17.0). Baseline characteristics of the cohort are represented in Table 1.

Baseline renal function differed substantially across early creatinine change groups, suggesting that apparent improvement may partly reflect regression from elevated admission values. This is further discussed in Section 4.4.

### 3.3. Distribution of Early Renal Function Patterns

Using KDIGO-based early pattern criteria between admission and 24–48 h, 470 (65.2%) patients were classified as stable, 162 (22.5%) were classed as early improvement, and 89 (12.3%) were classed as early deterioration.

Baseline renal function differed markedly across groups. Admission creatinine was highest in the improvement group (1.8 [1.2–2.7] mg/dL) compared with the deterioration (1.0 [0.6–1.9] mg/dL) and stable (0.8 [0.7–1.1] mg/dL; overall *p* < 0.001) groups. Consistently, admission eGFR was lowest in the improvement group (35.5 [21.1–55.0] mL/min/1.73 m^2^) versus the deterioration (62.0 [32.5–98.8]) and stable (89.0 [63.0–101.0]; overall *p* < 0.001) groups. Pre-existing CKD was more frequent in the deterioration and improvement groups (21.3% and 21.0%) than in the stable group (5.7%, *p* < 0.001).

### 3.4. Unadjusted Outcomes by Renal Function Group

Mortality differed significantly across early creatinine change groups (*p* = 0.007). Death occurred in 40/89 (44.9%) patients with early deterioration, in 78/162 (48.1%) with early improvement, and in 165/470 (35.1%) with stable renal function. Compared with the stable group, the unadjusted odds of death were higher for the improvement group (OR 1.72, 95% CI 1.20–2.46, Fisher *p* = 0.004) and numerically higher for the deterioration group (OR 1.51, 95% CI 0.95–2.39, Fisher *p* = 0.093).

Acute de novo hemodialysis also differed by early creatinine change group (*p* = 0.008), occurring in 2/89 (2.2%) of the deterioration group, 9/162 (5.6%) of the improvement group, and 6/470 (1.3%) of the stable group. Relative to the stable group, the improvement in early creatinine changes showed higher odds of acute hemodialysis (OR 4.55, 95% CI 1.59–12.99, Fisher *p* = 0.004), whereas deterioration was not significantly different (OR 1.78, 95% CI 0.35–8.95, Fisher *p* = 0.620).

Length of stay differed across groups (*p* = 0.007). Median hospitalization was 9.0 (5.0–15.0) days in the deterioration group versus 13.0 (8.0–17.8) in the improvement group and 13.0 (8.0–18.0) in the stable group.

In-hospital outcomes are represented in Table 2.

### 3.5. Multivariable Association with In-Hospital Mortality

In the primary multivariable logistic regression model (*n* = 713 complete cases for the selected covariates), using the stable group as reference, the early creatinine change category remained informative. Early improvement was independently associated with higher mortality (adjusted OR 1.53, 95% CI 1.03–2.28, *p* = 0.034), whereas early deterioration showed a non-significant directionally higher risk (adjusted OR 1.45, 95% CI 0.88–2.39, *p* = 0.148). Age was a strong independent predictor (per-year OR 1.05, 95% CI 1.03–1.07, *p* < 0.001). Treatment variables (favipiravir and antibiotic use) were also associated with higher mortality in this model, consistent with confounding by indication/severity.

In a secondary, severity-adjusted model restricted to patients with available pulmonary involvement and CRP (*n* = 364), pulmonary involvement was independently associated with mortality (per 1% increase OR 1.03, 95% CI 1.02–1.04, *p* < 0.001). In this restricted analysis, the early improvement in early creatinine change groups remained associated with increased mortality (adjusted OR 2.84, 95% CI 1.57–5.15, *p* < 0.001), while early deterioration was not statistically significant.

**Figure 2:**
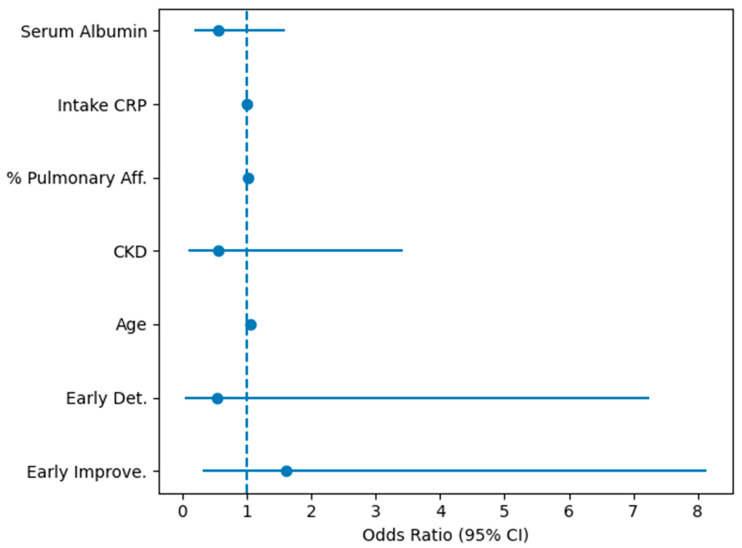
Forest plot of multivariable logistic regression analysis. Forest plot of multivariable logistic regression analysis evaluating factors associated with in-hospital mortality. Odds ratios (ORs) and 95% confidence intervals are shown for early renal function trajectories (Early Improve = early improvement, and Early Det. = early deterioration, with stable renal function as reference), age, CKD, % Pulmonary Aff. = % of affected pulmonary parenchyma, intake CRP, and serum albumin. The dashed vertical line indicates an odds ratio of 1.0.

The complete logistic regression can be better visualized in Figure 2.

Pulmonary involvement and CRP were not available for all patients due to incomplete imaging and laboratory testing during peak pandemic periods, resulting in a reduced sample size.

### 3.6. KDIGO-AKI Diagnosis Across Early Creatinine Change Groups

The recorded KDIGO-AKI diagnosis differed strongly across groups (*p* < 0.001), occurring in 31/89 (34.8%) in the early deterioration group, 73/162 (45.1%) in the early improvement group, and 72/470 (15.3%) in the stable group, supporting that early creatinine dynamics captured clinically meaningful renal risk phenotypes.

## 4. Discussions

### 4.1. Interpretation of Results

Our main finding is that early renal function dynamics within 24–48 h after admission carry clinically meaningful prognostic information but not always in the intuitive direction. Using KDIGO-based creatinine criteria, the cohort was separated into three early phenotypes (stable, early improvement, early deterioration), and these phenotypes differed not only by outcomes but also by baseline renal status and comorbidity burden. The stable group had the lowest mortality, while both early improvement and early deterioration clustered toward worse outcomes, with the early improvement group showing the most counterintuitive profile: despite “improving” creatinine, it carried higher mortality and higher AKI burden than the stable group. Importantly, early creatinine change patterns must be interpreted in the context of baseline renal function, as apparent improvement is largely driven by high admission creatinine and lower baseline eGFR.

This “improvement paradox” is best interpreted as a marker of high-risk baseline physiology rather than a protective signal. The early improvement group entered hospitalization with substantially higher admission creatinine and lower eGFR and a higher prevalence of CKD and cardiovascular comorbidity (Table 1). A rapid fall in creatinine over 24–48 h in such patients can reflect several high-risk situations: (i) admission coinciding with recovery from community-acquired AKI (e.g., dehydration, pre-renal azotemia, hemodynamic instability) rather than “true renal recovery”; (ii) hemodilution after aggressive fluid resuscitation; and/or (iii) “regression toward the mean” when baseline creatinine is markedly abnormal at presentation. This conceptual issue is specifically recognized in COVID-19: Wainstein et al. highlight that the traditional KDIGO approach may miss patients who are admitted during recovery, manifested by a decrease in serum creatinine, and they propose an extended definition precisely to capture this phenotype, supporting the idea that early creatinine decline can indicate preceding kidney injury, not benign physiology [24].

The early deterioration group, while smaller, also showed a high-risk pattern with increased mortality compared with stable renal function and a higher proportion of AKI. Mechanistically, early deterioration likely captures patients in whom COVID-19-related systemic disease is progressing quickly during the first 48 h, through hemodynamic stress, inflammatory injury, microvascular dysfunction, and respiratory-failure-associated renal congestion [25,26,27]. COVID-19 cohorts repeatedly show that AKI is tightly linked to illness severity and adverse outcomes and that dynamic kidney markers (creatinine/BUN trajectories) stratify severity and prognosis [12,28]. In parallel, ventilatory and hemodynamic factors (e.g., positive pressure ventilation effects on renal perfusion/venous congestion) are increasingly recognized as contributors to AKI in severe COVID-19, reinforcing why early in-hospital worsening can reflect rapidly escalating systemic stress [29].

A second key interpretative point is that our data suggest a U-shaped risk pattern around stable renal function, whereby any marked early creatinine change—whether improvement or deterioration—signals higher risk than biochemical stability. Clinically, this makes sense in a pandemic or postpandemic cohort. Stability within 48 h may represent patients with preserved renal reserve and less hemodynamic volatility early after admission. In contrast, early improvement may mark patients arriving in an unstable or under-resuscitated state (with subsequent correction), while early deterioration may mark those who declare multi-organ dysfunction early [30,31,32]. Importantly, the observation that “improving” looks prognostically worse than “stable” is not unique to our study conceptually: recovery patterns are often conditional on survival. Charytan et al. reported that kidney function frequently recovers among survivors, implying that those with early creatinine improvement are not necessarily “healthier” at baseline; rather, the relationship between recovery and mortality is confounded by severity and competing risk [28].

Finally, the multivariable model supports that trajectories are not purely epiphenomena. Even after adjustment for age, CKD, inflammatory burden (CRP), nutritional/physiologic reserve (albumin), and pulmonary involvement, the improvement in early creatinine change groups remained associated with higher odds of in-hospital mortality, while early deterioration showed a directionally similar but less precise association. This pattern is consistent with the clinical reality that “improvement” can represent a recovering injury state, not a normal state, especially when the admission value is already pathologic [33,34]. The key message for clinicians is therefore not that creatinine improvement is harmful but that a large early shift (in either direction) identifies patients whose renal status is unstable and who warrant closer surveillance and aggressive risk mitigation.

### 4.2. Comparison with the Current Literature

Our findings align with and extend the prior COVID-19 literature showing that early dynamic renal changes provide stronger prognostic information than static creatinine values or binary AKI classifications. Several large COVID-19 cohorts and meta-analyses established the baseline context: AKI is common in hospitalized COVID-19 and is linked to higher mortality, particularly in more severe disease and in patients with lower baseline kidney reserve [28,31]. We underline a creatinine-change-based lens in the first 48 h, which is less frequently operationalized as a primary exposure.

Regarding disease burden, a large comparative cohort analysis by Moledina et al. reported that COVID-19 was associated with high rates of AKI even after extensive adjustment, and AKI/dialysis clustered with markers of systemic severity [35]. Similar outcome patterns—AKI linked to high mortality and frequent need for RRT in severe presentations—appear in national or multi-hospital registry experiences, such as the Spanish FRA-COVID SEN registry data [36] and large single-center cohorts from settings with substantial comorbidity burdens [37].

While not all COVID-19 studies explicitly formalize “early creatinine change groups” in the first 48 h, multiple reports emphasize that early renal impairment (low eGFR/renal dysfunction at admission or early during hospitalization) predicts poor outcomes even outside ICU populations. For example, the eGFR-COV19 study showed that early reduction in eGFR among patients admitted to regular medical wards predicted poor outcome [38]. Other hospitalized-cohort analyses similarly link reduced eGFR/kidney injury markers to in-hospital mortality [39].

A key piece of the literature that aligns directly with our counterintuitive observation is the concept of “recovering AKI” at presentation. Wainstein and colleagues proposed an extended KDIGO definition that captures patients who are already improving (falling creatinine) early after admission—individuals who can be missed by standard creatinine-rise criteria but nonetheless have worse outcomes than patients without AKI [24]. Large-scale work on community-acquired AKI has explicitly operationalized AKI phenotypes that include early increase in creatinine during hospitalization as evidence of kidney injury present at admission [40].

In critically ill populations, the link between baseline kidney function and recovery/nonrecovery has also been demonstrated. In STOP-COVID (patients with COVID-19 AKI treated with dialysis), lower baseline eGFR was strongly associated with kidney nonrecovery [41]. This helps rationalize why an early improvement group, which entered with poorer renal function, can still carry high mortality despite apparent biochemical improvement. Analyses involving dialysis initiation are exploratory and underpowered, and adjusted estimates should be interpreted with caution.

### 4.3. COVID-19-Specific Pathophysiological Considerations

Beyond baseline comorbidity and illness severity, several COVID-19-specific biological mechanisms may plausibly explain why early renal trajectories behave in a counterintuitive manner, particularly the adverse profile of the “early improvement” group.

At a molecular and metabolic level, SARS-CoV-2 infection induces a profound systemic inflammatory and immunometabolic response that directly affects renal tubular cells [42,43]. Single-cell and autopsy studies have shown that proximal tubular epithelial cells exhibit mitochondrial dysfunction, altered fatty acid oxidation, and downregulation of energy-dependent transporters, even in the absence of an overt creatinine rise, predisposing to transient or recovering AKI phenotypes rather than progressive injury [44,45,46]. In this context, a rapid fall in creatinine may reflect partial restoration of glomerular filtration while subcellular metabolic injury persists, decoupling biochemical improvement from true organ recovery.

COVID-19 is also characterized by endothelial dysfunction and microvascular injury, driven by cytokine excess, complement activation, and dysregulated coagulation [47,48,49]. Renal peritubular capillary rarefaction and microthrombi have been consistently described, leading to heterogeneous and dynamic perfusion states within the kidney [50,51,52]. These mechanisms support why both improving and deteriorating creatinine trajectories may converge toward similar outcomes: early fluctuations can reflect shifting renal perfusion and filtration rather than stable recovery.

Importantly, SARS-CoV-2-associated AKI often overlaps with systemic metabolic stress, including insulin resistance, catabolic state, and hypoalbuminemia, which independently modulate creatinine generation and distribution volume [53,54,55,56]. Reduced muscle creatinine production during acute illness can contribute to an apparent “improvement” in serum creatinine without a parallel improvement in renal cellular integrity [50,55,57,58,59]. This phenomenon is particularly relevant in older, comorbid populations and may amplify the observed dissociation between early biochemical trends and clinical outcomes.

### 4.4. Limitations

Several limitations should be acknowledged. First, the retrospective, single-center design limits causal inference and introduces the possibility of residual confounding, despite multivariable adjustment. Important indicators of acute illness severity, such as detailed respiratory parameters, hemodynamic data, urine output, and fluid balance, were not available and could have influenced early creatinine dynamics. Additionally, body mass index and formal obesity categories were not consistently available in the electronic records and could not be included as covariates, although obesity may influence both respiratory severity and renal hemodynamics in COVID-19. The lack of fluid balance data limits interpretation of dilutional effects on creatinine.

Secondly, renal function was assessed using only two early time points, which limits the ability to capture true renal dynamics and increases sensitivity to transient hemodilution or laboratory variability (admission and 24–48 h). In acute COVID-19, serum creatinine is affected by fluid shifts, catabolic state, and reduced creatinine generation; therefore, early “improvement” may not reflect true renal recovery, and early “deterioration” may encompass heterogeneous pathophysiological processes. The lack of more frequent early measurements limited modeling of finer early creatinine changes. Exclusion of patients without repeated creatinine measurements may have introduced selection bias, particularly if early mortality was overrepresented in this group. Also, the absence of pre-admission creatinine prevented distinction between community-acquired and hospital-acquired AKI.

Third, AKI classification relied primarily on serum creatinine, as urine output criteria were not systematically captured. Chronic kidney disease was recorded as a binary variable without staging, which may have introduced heterogeneity within early creatinine change groups, particularly among patients with impaired baseline renal reserve. Creatinine was the sole renal marker available across the cohort; given its dependence on muscle mass and metabolism, subclinical kidney injury may have been underestimated.

Fourth, inclusion in the analytic cohort required complete early creatinine data, which may have introduced selection bias if missingness was related to disease severity, early death, or health system strain during pandemic surges. In addition, treatment variables reflect real-world care under rapidly evolving COVID-19 protocols and are subject to confounding by indication. Inclusion of patients surviving to 24–48 h may introduce survivor bias.

In addition, CKD was captured as a binary variable rather than by stage; therefore, a single CKD category may not fully reflect baseline renal functional heterogeneity or renal reserve across patients.

In patients with very high admission creatinine values, early decreases may reflect regression from extreme values rather than true functional improvement.

Finally, outcomes were restricted to in-hospital events. Long-term renal recovery, post-discharge dialysis dependence, and late mortality could not be assessed, although these outcomes are highly relevant in COVID-19-associated kidney injury. Analyses involving radiologic lung damage were secondary and conducted in a reduced sample, which may limit estimate stability and requires cautious interpretation. Analyses involving dialysis initiation are exploratory and underpowered, and adjusted estimates should be interpreted with caution.

### 4.5. Future Perspectives

Future research should aim to prospectively validate early renal creatinine change phenotypes and disentangle biochemical change from true renal recovery. Studies with dense early sampling (e.g., serial creatinine within the first 72 h, urine output, and fluid balance) and time-resolved severity markers would allow for more precise modeling of early creatinine changes and help distinguish hemodilution or creatinine kinetics from structural kidney injury. Incorporating baseline pre-admission creatinine and CKD staging would further refine risk stratification.

A second priority is the integration of kidney injury and stress biomarkers that reflect tubular damage and cellular stress independent of filtration, such as NGAL, KIM-1, TIMP-2·IGFBP7, and markers of endothelial injury. These biomarkers have shown prognostic value in AKI and may clarify why apparent early creatinine improvement can coexist with adverse outcomes, particularly in inflammatory states like COVID-19 [9,60,61,62]. Coupling biomarkers with early creatinine changes analysis could identify patients with “biochemical recovery but biological injury.

Third, multisystem phenotyping is needed. COVID-19 highlights the kidney’s sensitivity to cardio-pulmonary interactions, endothelial dysfunction, and immunometabolic stress. Future models should integrate renal trajectories with respiratory mechanics, venous congestion indices, inflammatory profiles, and nutritional status, enabling early, bedside risk tools that move beyond single-organ metrics [63,64].

Finally, extending follow-up beyond hospitalization is essential. Linking early in-hospital renal trajectories to post-discharge outcomes—CKD progression, dialysis dependence, cardiovascular events, and mortality—would establish whether the “early improvement paradox” predicts long-term vulnerability. Such evidence could inform targeted surveillance and post-acute care pathways for patients whose early creatinine changes signal hidden risk rather than recovery.

## 5. Conclusions

In hospitalized patients with COVID-19, early renal function trajectories within the first 24–48 h provide prognostic information beyond static creatinine values. Using KDIGO-based criteria, we found that patients with stable renal function had the most favorable in-hospital outcomes, whereas both early deterioration and the seemingly paradoxical early improvement trajectories were associated with higher mortality and greater renal risk. These findings underscore that early creatinine dynamics should not be interpreted in isolation, as apparent improvement may reflect recovery from a preceding kidney insult, hemodynamic instability, or disease-related metabolic effects rather than true renal recovery.

Our results highlight the importance of early creatinine change-based renal assessment in COVID-19 and suggest that short-term creatinine changes—particularly rapid early improvement—may identify a subgroup of patients with heightened vulnerability who warrant closer monitoring and intensified supportive care.

Early changes in creatinine should be interpreted as prognostic signals rather than indicators of renal recovery, emphasizing their value for risk stratification rather than causal inference.

Future prospective studies incorporating granular physiologic data, kidney-specific biomarkers, and long-term follow-up are needed to validate these phenotypes and determine how early renal creatinine change patterns can be integrated into clinical risk stratification and post-acute management strategies.

## Figures and Tables

**Table 1:** Baseline characteristics of the study population.

Variable	Overall (*n* = 721)	Stable (*n* = 470)	Early Improvement (*n* = 162)	Early Deterioration (*n* = 89)	*p*-Value
Age, years	68.0 ± 12.6	66.9 ± 12.4	69.8 ± 12.3	71.2 ± 13.1	0.002
Male sex, *n* (%)	398 (55.2)	250 (53.2)	96 (59.3)	52 (58.4)	0.41
Chronic kidney disease, *n* (%)	80 (11.1)	27 (5.7)	34 (21.0)	19 (21.3)	<0.001
Hypertension, *n* (%)	432 (59.9)	265 (56.4)	107 (66.0)	60 (67.4)	0.02
Diabetes mellitus, *n* (%)	228 (31.6)	137 (29.1)	57 (35.2)	34 (38.2)	0.08
Coronary artery disease, *n* (%)	198 (27.5)	114 (24.3)	55 (34.0)	29 (32.6)	0.01
Atrial fibrillation, *n* (%)	96 (13.3)	52 (11.1)	28 (17.3)	16 (18.0)	0.03
Pulmonary involvement, %	35 (20–55)	30 (15–50)	45 (30–65)	50 (35–70)	<0.001
Creatinine at admission, mg/dL	0.9 (0.7–1.4)	0.8 (0.7–1.1)	1.8 (1.2–2.7)	1.0 (0.6–1.9)	<0.001
eGFR at admission, mL/min/1.73 m^2^	72 (45–94)	89 (63–101)	35 (21–55)	62 (33–99)	<0.001
Urea at admission, mg/dL	48 (32–78)	41 (30–64)	78 (51–120)	56 (34–93)	<0.001
Hemoglobin, g/dL	12.8 ± 2.1	13.1 ± 2.0	12.3 ± 2.2	12.4 ± 2.3	<0.001
Albumin, g/dL	3.4 ± 0.6	3.5 ± 0.5	3.2 ± 0.6	3.3 ± 0.6	<0.001
CRP at admission, mg/L	62 (28–118)	54 (24–98)	81 (41–156)	75 (36–139)	<0.001
Antiviral therapy, *n* (%)	412 (57.1)	256 (54.5)	103 (63.6)	53 (59.6)	0.09
Antibiotic therapy, *n* (%)	578 (80.2)	360 (76.6)	146 (90.1)	72 (80.9)	<0.001

Data are presented as mean ± SD, median (IQR), or number (percentage), as appropriate. Early renal function trajectories were defined using KDIGO-based criteria applied to serum creatinine changes between admission and 24–48 h. *p*-values refer to comparisons across early creatinine change groups.

**Table 2:** In-hospital outcomes according to early renal function pattern.

Outcome	Overall (*n* = 721)	Stable (*n* = 470)	Early Improvement (*n* = 162)	Early Deterioration (*n* = 89)	Unadjusted OR vs. Stable (95% CI)	*p*-Value
Mortality, *n* (%)	283 (39.3)	165 (35.1)	78 (48.1)	40 (44.9)	Improvement: 1.72 (1.20–2.46)	0.004
					Deterioration: 1.51 (0.95–2.39)	0.093
Acute kidney injury (KDIGO), *n* (%)	176 (24.4)	72 (15.3)	73 (45.1)	31 (34.8)	Improvement: 4.55 (3.05–6.78)	<0.001
					Deterioration: 2.94 (1.77–4.89)	<0.001
Acute de novo hemodialysis, *n* (%)	17 (2.4)	6 (1.3)	9 (5.6)	2 (2.2)	Improvement: 4.55 (1.59–12.99)	0.004
					Deterioration: 1.78 (0.35–8.95)	0.620
Length of hospital stay, days	13 (7–17)	13 (8–18)	13 (8–18)	9 (5–15)	-	0.007

Data are presented as median (IQR) or number (percentage). Odds ratios (ORs) are unadjusted and calculated using the stable renal function group as the reference. AKI was defined according to KDIGO criteria. *p*-values refer to overall group comparisons or unadjusted logistic regression, as appropriate.

## Data Availability

All the data and materials will be made available on written request.

## Abbreviations

AKI	Acute kidney injury
ARDS	Acute respiratory distress syndrome
BUN	Blood urea nitrogen
CI	Confidence interval
CKD	Chronic kidney disease
CKD-EPI	Chronic Kidney Disease Epidemiology Collaboration
COVID-19	Coronavirus disease 2019
CRP	C-reactive protein
CV	Cardiovascular
eGFR	Estimated glomerular filtration rate
GDPR	General Data Protection Regulation
GFR	Glomerular filtration rate
ICU	Intensive care unit
IQR	Interquartile range
KDIGO	Kidney Disease: Improving Global Outcomes
KIM-1	Kidney injury molecule-1
LOS	Length of stay
MODS	Multiple organ dysfunction syndrome
NGAL	Neutrophil gelatinase-associated lipocalin
OR	Odds ratio
PEEP	Positive end-expiratory pressure
RRT	Renal replacement therapy
SD	Standard deviation
SARS-CoV-2	Severe acute respiratory syndrome coronavirus 2
STROBE	Strengthening the Reporting of Observational Studies in Epidemiology
TIMP-2·IGFBP7	Tissue inhibitor of metalloproteinases-2 × insulin-like growth factor-binding protein 7
